# Non-genetic inactivation of caspase-3 and P53 increases cancer cell fitness by PDIA4 redistribution

**DOI:** 10.1038/s41388-025-03606-7

**Published:** 2025-10-21

**Authors:** Gal Twito, Faiza Amterat Abu Abayed, Ayelet Gilad, Suma Biadsy, Noa Gavriel, Suad Sheikh Suliman, Yarden Mizrahi, Hila Megged, Mor Tenenboim, Naim Abu-Freha, Aeid Igbaria

**Affiliations:** 1 Department of Life Sciences, Ben-Gurion University of the Negev, Beer Sheva, Israel; 2 Institute of Gastroenterology and Liver Diseases, Soroka Medical Center, Faculty of Health Sciences, Ben Gurion University of the Negev, Beer Sheva, Israel

**Keywords:** Apoptosis, Stress signalling

## Abstract

Numerous cellular pathways are known to cause resistance in cancer cells. The unfolded protein response (UPR), a signaling pathway activated during proteostasis stress in the endoplasmic reticulum (ER), is an adaptive process to increase cancer cell fitness. However, the molecular mechanism between ER stress, UPR activation, and chemoresistance is insufficiently understood. Here, we report that ER stress induction and UPR activation are necessary for chemoresistance to cisplatin and doxorubicin. Mild ER stress is a sufficient precondition for cancer cells to evade cisplatin- and doxorubicin-associated cell death. Mechanistically, ER stress induction results in the redistribution of PDIA4 from the ER to the cytosol, facilitated by the c-tail-anchored proteins DNAJB12 and DNAJB14 and the cytosolic HSC70-cochaperone SGTA. In the cytosol, PDIA4 forms an inhibitory interaction with caspase-3 and wt-p53, leading to their attenuation and increased cancer cell proliferation. Furthermore, we show that PDIA4 must originate from the ER to inhibit caspase-3 and wt-p53 in the cytosol. Silencing PDIA4, DNAJB12/14, or SGTA rescues wt-p53 and caspase-3 activity. Finally, we found that in tumors isolated from colorectal cancer patients, PDIA4 and DNAJB12 are highly expressed compared to their healthy tissues; this expression is associated with the induction of the UPR. Our data show a novel non-genetic mechanism to inhibit apoptosis and suggest PDIA4, DNAJB12/14, and SGTA as novel therapeutic targets to rescue apoptosis and inhibit proliferation in cancer cells.

## Introduction

Chemoresistance arising from genetic or epigenetic changes poses a significant challenge in cancer treatment and is responsible for poor prognosis and survival outcomes [1–4]. The genotoxic drugs cisplatin and doxorubicin are used to treat various cancer types, including lung, breast, ovarian, and colorectal cancers, among others. The drugs increase cytotoxicity by causing DNA damage, leading to cell death [5–7]. Additional cellular pathways were reported to contribute to the toxicity/resistance of cisplatin and doxorubicin, including the disruption of proteostasis in the endoplasmic reticulum (ER) [1, 8–11].

The ER is responsible for the synthesis and proper folding of approximately one-third of all cellular proteins. When the folding demand surpasses the ER’s capacity, proteostasis is disrupted, resulting in a condition known as ER stress. In response, cells initiate the unfolded protein response (UPR), an adaptive signaling pathway mediated by three ER-resident transmembrane sensors: protein kinase RNA-like ER kinase (PERK), activating transcription factor 6 (ATF6), and inositol-requiring enzyme 1 (IRE1). These sensors detect the accumulation of misfolded proteins through their luminal domains and trigger downstream pathways that enhance folding capacity and restore homeostasis. Persistent UPR activation, however, is frequently observed in cancer and contributes to malignancy, metastasis, chemoresistance, invasion, and other pro-tumorigenic processes [12–16].

The role of the UPR in chemoresistance is complex and context-dependent, acting as a double-edged sword. On one hand, cisplatin-induced UPR activation has been associated with enhanced cell death; on the other hand, numerous studies suggest that the UPR can adopt a cytoprotective role, promoting cell survival and contributing to chemoresistance. Inhibition of the UPR has been shown to sensitize cancer cells to a variety of chemotherapeutic agents. Conversely, other studies have demonstrated that chemotherapy-induced UPR activation is essential for effective cancer cell killing. Therefore, the role of the UPR in modulating cancer cell responses to chemotherapy remains controversial, and the underlying reasons for these discrepancies are not yet fully understood [15, 17–22].

Recently, we and others reported a novel ER stress-induced surveillance mechanism by which proteins from the ER are refluxed to the cytosol to inhibit proapoptotic pathways. This conserved mechanism, called ERCYS (ER to cytosol signaling), is associated with gain of function of the refluxed proteins and increased cancer cell fitness. ERCYS is constitutively active in human tumors and is mediated by chaperones and cochaperones from the ER and the cytosol, namely DNAJB12, DNAJB14, HSC70, and SGTA [23–29].

Cisplatin and doxorubicin treatment activate ER stress and the UPR through an unspecified mechanism [30, 31]. In addition, the relationship between ER stress, UPR activation, and chemoresistance remains unclear [7, 13–16, 20, 32–34]. Here, we found that cancer chemoresistance is associated with the decreased activities of wt-p53 and caspase-3, and the increased ER stress signaling and UPR activation. We also show that preconditioning cells with mild ER stress is sufficient to promote chemoresistance. Mechanistically, ERCYS allows a subset of ER proteins to reflux to the cytosol facilitated by DNAJB12/14 and SGTA. In the cytosol, PDIA4 originating from the ER binds and inhibits wt-p53 and caspase3 activity. This non-genetic resistance can be reversed by silencing PDIA4 or inhibiting its cytosolic accumulation by targeting DNAJB12, DNAJB14, and SGTA.

## Material and methods

### Cell culture and transfection

Human A549 and MCF-7 cell lines were cultured in Dulbecco’s modified Eagle’s medium (DMEM) supplemented with 10% FBS and 1% antibiotics at 37 °C in a 5% CO2 incubator. Cells were transfected with Lipofectamine 2000 (Thermo Fisher Scientific) according to the manufacturer’s protocols. Thapsigargin (Tg), Cisplatin (Cis), and Doxorubicin (Dox) were purchased from Sigma-Aldrich. Tunicamycin (Tm) was purchased from Calbiochem.

### XTT assay and caspase 3/7 assay

The Caspase-Glo® 3/7 kit was purchased from Promega. The caspase 3/7 assay was done according to the manufacturer’s instructions. In brief, 100 μl of Caspase-Glo® 3/7 reagent was added to each well in 96 well plates and mixed thoroughly. After 3 h of incubation at room temperature, the luminescence of each sample was measured using a Varioskan Lux microplate reader (ThermoFisher Scientific). Cell proliferation assay was done using the XTT kit (BioInd, SARTORIUS). 50 μL of XTT reagent solution/activation mix was added to 2000 cells grown in 96 wells and incubated for 4 h at 37 °C. Absorbance was then measured at 450 and 690 nanometers.

### Western blotting

The growth medium was collected, centrifuged, and washed with an ice-cold PBS buffer before being pelleted again. The attached cells were washed with PBS, and the lysates were collected using RIPA buffer (25 mM Tris/HCl pH 7.5, 150 mM NaCl, 1%sodium deoxycholate, 0.1% SDS, and 1% NP-40). The pelleted medium and the cell lysates were combined and centrifuged at 4 °C for 10 min at 11,000 × *g*. Equal proteins were then loaded on SDS/PAGE. Diluted primary antibodies (1:1000) were incubated overnight at 4 °C. After three washes, membranes were incubated with fluorescent secondary antibodies (1:10,000 dilution) and incubated for 1 h before scanning using iBright Imaging Systems (Thermofisher). All antibodies used in this study are listed in Supplementary Table S2.

### RNA extraction and real-time PCR (qPCR)

NucleoSpin RNA Mini kit (Macherey Nagel) was used for RNA isolation from A549 and MCF-7 cells. 500–1000 ng of total RNA was used for cDNA synthesis using Maxima™ Reverse Transcriptase (ThermoFisher). The qPCR reaction was done using qPCR SyGreen (BioSystems) using Quantstudio 1 (Applied Biosystems). Relative mRNA levels and gene expression levels were normalized to GAPDH or HPRT1. All Primer sequences were used as described in [35].

### XBP1 splicing assay

XBP1 mRNA splicing was assessed by PCR using primers flanking the 26-nucleotide intron to amplify both spliced and unspliced forms. The PCR was performed using a standard PCR protocol: initial denaturation at 95 °C for 5 min, followed by 30 cycles of 95°C for 30 s, 60°C for 30 s, and 72°C for 40 s, with a final extension at 72 °C for 7 min. The primers used were: forward hXBP1: 5ʹ-GGAGTTAAGACAGCGCTTGGGGA -3ʹ and reverse hXBP1: 5ʹ- TGTTCTGGAGGGGTGACAACTGGG -3ʹ. PCR products were separated on 3.5% agarose gels and stained with ethidium bromide.

### Subcellular protein fractionation

The protocol was described earlier in [36]. Cells were washed with ice-cold PBS and then trypsinized for 2 min. Cells were collected and pelleted at 100RCF for 5 min at 4 °C. Pellets were then washed and resuspended in digitonin buffer (50 mM HEPES pH 7.4, 150 mM NaCl, 10 μg/ml digitonin (25 μg/mL)). After 10 min of incubation at 4 °C, cells were pelleted at 2000RCF for 5 min at 4°C. The supernatant at this stage is collected as the cytosolic fraction. The remaining pellet was dissolved in NP40 buffer (50 mM HEPES pH 7.4, 150 mM NaCl, 1% NP40). After 30 min incubation on ice, cells were centrifuged at 7000RCF for 5 min at 4 °C. The supernatant at this stage is collected as the membranal fraction.

### Immunoprecipitation

Immunoprecipitation assay was done using the protocol published in [24]. Cells were collected in Co-Immunoprecipitation buffer (50 mM Tris/HCl pH 8, 150 mM NaCl, 0.5% TritonX100, and 1 mM EDTA) and incubated for 30 min on ice. After incubation, the lysate was cleared by 10 min of centrifugation at 11,000 × *g* at 4 °C. Equal amounts of proteins were taken for each IP and incubated overnight with different primary antibodies at (1 μg Ab/1000 μg protein) at 4°C. Dynabeads protein A (Life Technologies) were added to the protein/antibodies mixture for 3 h at 4°C with rotation. After the incubation, the beads were washed and eluted with 50 μl of Laemmli sample buffer, heated for 5 min at 70 °C, and loaded to SDS/PAGE.

### Tissue handling

Human samples used for the analyses shown in this manuscript were provided by the the Colorectal cancer (CRC) biobank in Soroka, Beer Sheva. the samples were collected after Helsinki approval of biobank samples collection, approval number 0137-18. Total RNA and protein lystaes were isolated using TRIzol Reagent following the manufactural instructions (Invitrogen).

### Statistical analysis

All experiments were done in biological triplicates. Data were analyzed using GraphPad Prism software. Unless otherwise specified, datasets were normally distributed and are presented as mean ± SD. Comparisons between groups were performed using unpaired, two-tailed Student’s t tests. Statistical significance was defined as p < 0.05. (***p < 0,001, **p < 0,01, *<0.05).

### Immunofluorescence assay

A total of 15,000 A549 cells were seeded in μ-Slide 8-well plates (Ibidi, Cat. 80826). Cells were fixed with 4% paraformaldehyde and permeabilized with 0.3% Triton X-100 in PBS. After washing, cells were blocked with 3% BSA in PBS for 1 h. Primary antibodies against PDIA4 (ERp72, Cat. 14712), Caspase-3 (Caspase-3/P17/P19, Cat. 68773), PDIA3 (ERp57/ERp60, Cat. 15967), and Calnexin (Cat. 10427) (Proteintech Group, Rosemont, IL, USA), and GAPDH (sc-47724, Santa Cruz Biotechnology, Dallas, TX, USA) were diluted 1:300 in PBS containing 0.1% Triton X-100 and 1% BSA, and incubated overnight at 4 °C. Cells were washed three times with PBS and incubated with secondary antibodies (1:400 in PBS with 1% BSA and 0.1% Triton X-100) for 1 h at room temperature. Secondary antibodies included Alexa Fluor™ 488-conjugated goat anti-mouse IgG (A-11001) and Alexa Fluor™ 633-conjugated goat anti-rabbit IgG (A-21070) (Invitrogen, Thermo Fisher Scientific). Following three PBS washes, cells were imaged using a Zeiss LSM 880 confocal laser scanning microscope with ZEN Lite 3.11 software at 63× magnification with oil immersion.Scale bar 10uM

## Results

### Generating MCF-7 and A549-CHEMORESISTANT CELLS

**Fig. 1:**
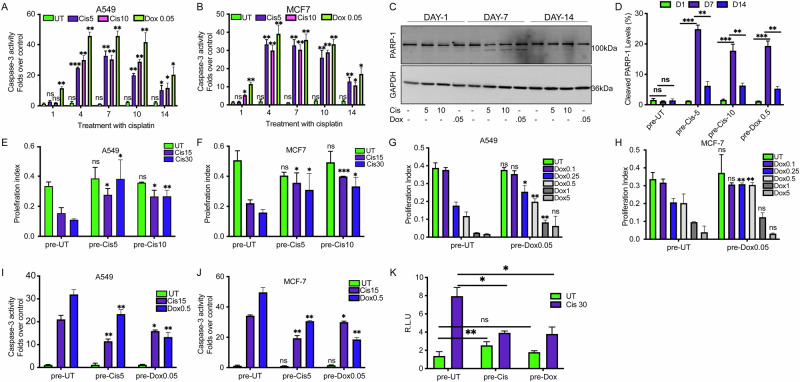
Cancer chemoresistance is associated with inhibition of caspase 3/7 and wt-p53 activities. **A**, **B** Folds change in Caspase3/7 activity after treatment with the indicated concentrations [μMs] of cisplatin (cis) or doxorubicin (dox) for 14 days in A549 and MCF-7 cell lines, respectively N = 3. **C** Representative immunoblot of PARP-1 and GAPDH in A549 cells treated for 1, 7, and 14 days post with cisplatin and doxorubicin at the indicated concentrations [μMs]. N = 3. **D** Quantification of cleaved PARP-1 levels as shown in (**C**). **E**, **F** XTT assays in cisplatin-pretreated cells challenged with high concentrations [μMs] of cisplatin as indicated in A549 and MCF-7 cell lines, respectively N = 3. **G**, **H** XTT assays in doxorubicin-pretreated cells challenged with high concentrations [μMs] of doxorubicin as indicated in A549 and MCF-7 cell lines respectively N = 3. **I–J** Folds changes of Caspase3/7 activity after treatment with the indicated concentrations [μMs] of cisplatin (cis) or doxorubicin (dox) in pretreated A549 and MCF-7 cell lines, respectively N = 3. **K** pre-UT, pre-cis5μM, and pre-dox 0.05 μM cells were transfected with the p53-luciferase construct for 24 h; then cells were treated with the indicated cisplatin and doxorubicin concentrations, and luciferase experiments were performed. All experiments were done in biological triplicates. (***p < 0,001, **p < 0,01, *<0.05).

Initially, we developed a workflow to select cells that survive an extended treatment of low concentrations of cisplatin and doxorubicin. We cultured A549 and MCF-7 cell lines with low concentrations of cisplatin and doxorubicin until confluency. Then, cells were diluted and re-treated with the same drug dosage (Fig. S1A). Two weeks later, the surviving cells were collected and subjected to high doses of cisplatin or doxorubicin (Fig. S1). We then tested the caspase3/7-dependent apoptosis in the surviving cells by analyzing caspase-3/7 activity and poly [ADP-ribose] polymerase 1 (PARP-1) cleavage after one, four, seven, ten, and fourteen days (Fig. S1A). Initially, PARP-1 cleavage was observed at low levels, particularly on day seven (Fig. 1A–D). Caspase-3 activity peaked at day seven for cisplatin-treated cells and decreased between days ten and fourteen (Fig. 1A, B). In doxorubicin-treated cells, caspase-3/7 activity peaked on day four and decreased between day seven and fourteen (Fig. 1A, B). High-concentration cisplatin (10μM) peaked on day four and decreased on day fourteen (Fig. 1A, B). Under those conditions, PARP-1 cleavage peaked on day seven and decreased by day fourteen (Fig. 1C, D). Based on these data, we chose the fourteen-day timepoint at which many cells resist cisplatin and doxorubicin treatment by decreasing the caspase-3-dependent apoptosis.

After two weeks of pretreatment with low concentrations of cisplatin, A549 and MCF-7 cells displayed increased proliferation compared to untreated cells when subsequently exposed to cytotoxic doses of cisplatin (Fig. 1E, F). A similar effect was observed with doxorubicin: cells pretreated with low doses showed a significantly higher proliferation index upon challenge with higher doxorubicin concentrations, relative to untreated controls (Fig. 1G, H). In both cases, caspase-3/7 activity was reduced in pretreated cells following toxic drug exposure, suggesting that suppression of apoptosis contributes to the observed increase in resistance to cisplatin and doxorubicin (Fig. 1I, J).

While wild-type p53 transcriptional activity was elevated in cisplatin-pretreated cells, its induction following treatment was markedly reduced in both cisplatin- and doxorubicin-pretreated cells compared to untreated controls (Fig. 1K). These observations suggest that prolonged exposure to low doses of chemotherapeutic agents promotes the acquisition of multiple survival mechanisms, including caspase-3/7-dependent apoptosis suppression and diminished activation of proapoptotic p53 signaling, ultimately enhancing both proliferation and chemoresistance.

Moreover, cells pretreated with one chemotherapeutic agent developed cross-resistance to others: cells pretreated with doxorubicin exhibited increased survival when challenged with cisplatin, and conversely, cisplatin-pretreated cells showed increased survival following doxorubicin treatment. Indicating that this adaptive response is not drug-specific but represents a broader survival strategy driven by apoptosis inhibition and impaired p53 function (Fig. 1I–K). Clonogenic survival assays corroborated these findings, showing markedly enhanced colony formation in cells pretreated with either cisplatin or doxorubicin compared to untreated controls (Figs. S1B, S1C). Thus, pretreatment with low concentrations of cisplatin or doxorubicin reprograms cancer cells toward an adaptive, chemoresistant state through modulation of apoptosis and p53 signaling pathways.

### Chemoresistant cancer cells induce the UPR

We then sought to investigate common factors altered during cisplatin and doxorubicin treatments that could explain the increased resistance in A549 and MCF-7 cells. Initially, we examined the role of the UPR in chemoresistance by focusing on the ER stress sensor IRE1α. Upon activation, IRE1α catalyzes the unconventional splicing of XBP1 mRNA to generate the transcription factor XBP1s, which upregulates genes that enhance ER protein folding and quality control. Under prolonged or severe stress, however, IRE1α becomes hyperactivated and broadens its RNase activity to initiate Regulated IRE1α-Dependent Decay (RIDD), selectively degrading mRNAs and precursor microRNAs to reduce ER load [35]. To evaluate both branches of IRE1α activity, we measured XBP1 splicing as a marker of transcriptional UPR activation, and assessed RIDD activity by monitoring the expression of established RIDD targets, including BLOC1S and SCARA3. The downregulation of these transcripts reflects elevated RIDD activity and provides additional insight into the extent and nature of ER stress signaling in our system [24, 25, 37].

**Fig. 2:**
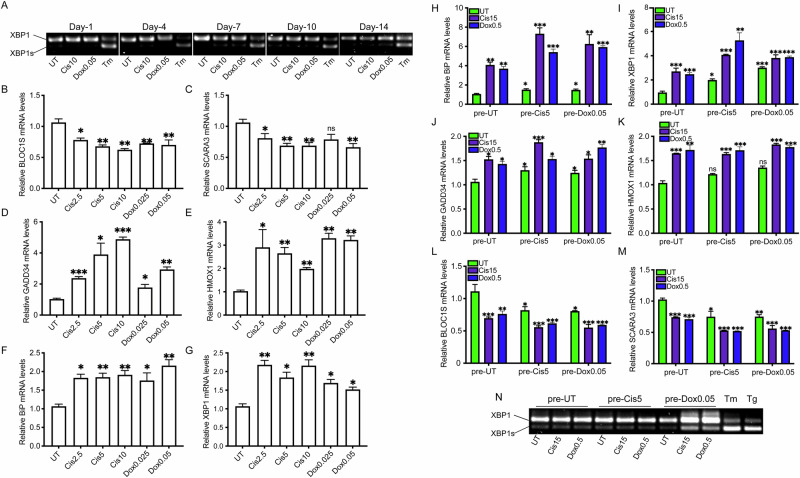
The Unfolded protein response (UPR) is constitutively active in chemoresistant cells. **A** Representative EtBr-stained agarose gel of XBP1 cDNA amplicons in different days post-treatment with the indicated concentrations [μMs] of cisplatin (Cis), doxorubicin (Dox), or Tunicamycin (Tm 250 ng/mL) in A549 cells. N = 3. **B**, **C** qPCR of relative mRNA levels of the IRE1-regulated genes BLOC1S and SCARA3 in A549 cells treated with different concentrations [μMs] of cisplatin (Cis) or doxorubicin (Dox) for 14 days N = 3. **D**, **E** qPCR of relative mRNA levels of the PERK-regulated genes GADD34 and HMOX1 in A549 cells treated with different concentrations [μMs] of cisplatin (Cis) or doxorubicin (Dox) for 14 days N = 3. **F**, **G** qPCR of relative mRNA levels of the ATF6-regulated genes BIP and total XBP1 in A549 cells treated with different concentrations [μMs] of cisplatin (Cis) or doxorubicin (Dox) for 14 days N = 3. **H**, **I** qPCR of relative mRNA levels of the ATF6-regulated genes BIP and total XBP1 in A549 cells treated with different concentrations [μMs] of cisplatin after pretreatment with either cisplatin or doxorubicin. N = 3. **J**, **K** qPCR of relative mRNA levels of the PERK-regulated genes GADD34 and total HMOX1 in A549 cells treated with different concentrations [μMs] of cisplatin after pretreatment with either cisplatin or doxorubicin. N = 3. **L**, **M** qPCR of relative mRNA levels of the IRE1-regulated genes BLOC1S and total SCARA3 in A549 cells treated with different concentrations [μMs] of cisplatin after pretreatment with either cisplatin or doxorubicin. N = 3. **N** Representative EtBr-stained agarose gel of XBP1 cDNA amplicons in pretreated-A549 cells challenged with cisplatin and doxorubicin at the indicated concentrations [μMs]. Tm (250 ng/mL) and Tg (15 nM) were used as positive controls. N = 3. All experiments were done in biological triplicates. (***p < 0,001, **p < 0,01, *<0.05).

Pretreatment with low doses of cisplatin or doxorubicin did not alter XBP1 splicing over the 14-day period. As a control, treatment of A549 cells with the N-linked glycosylation inhibitor tunicamycin (Tm) induced robust XBP1 splicing, confirming assay sensitivity. In contrast, BLOC1S and SCARA3 mRNA levels were significantly reduced in both A549 and MCF-7 cells relative to untreated controls (Fig. 2A–C and S2A–S2C). To determine whether this downregulation was dependent on IRE1 activity, we used MKC-3946, a selective inhibitor of IRE1 RNase function. Cisplatin pretreatment alone significantly decreased BLOC1S and SCARA3 expression, whereas cotreatment with MKC-3946 restored transcript levels to those of control cells. These results indicate that selective activation of the IRE1α–RIDD pathway mediates the downregulation of specific mRNAs and may contribute to enhanced cellular fitness under chronic low-dose chemotherapeutic stress (Fig. S2C).

Next, we assessed activation of the PERK arm of the UPR by examining the mRNA levels of *HMOX1* and *GADD34*, two established downstream targets of PERK signaling. In both A549 and MCF-7 cells, two-week pretreatment with varying concentrations of cisplatin or doxorubicin resulted in a robust induction of *HMOX1* and *GADD34* expression (Fig. 2D–E and S2D–S2F). This effect was dependent on PERK activity, as pharmacological inhibition with GSK2606414 prevented the increase, with cotreatment restoring *HMOX1* and *GADD34* transcript levels to those observed in untreated controls. We then evaluated the contribution of ATF6 signaling by analyzing the expression of *BIP* and total *XBP1*, two genes transcriptionally regulated by ATF6. In both A549 and MCF-7 cells, pretreatment with cisplatin or doxorubicin induced marked upregulation of *BIP* and *XBP1* relative to untreated controls, consistent with ATF6 activation (Fig. 2F–G and S2G–S2I). Inhibition of ATF6 activity with Ceapin-A7 abolished this effect, as cotreatment restored *BIP* and *XBP1* transcript levels to baseline (Fig. S2I). Taken together, these data demonstrate that prolonged pretreatment with low-dose chemotherapeutic agents engages IRE1, PERK, and ATF6 signaling, and that activation of these stress-adaptive pathways is a prominent feature of pretreated cells that may contribute to the development of chemoresistance.

To investigate the role of UPR activation in chemoresistance, chemoresistant A549 and MCF-7 cells were re-cultured for 24 h to allow recovery before re-exposure to high concentrations of doxorubicin or cisplatin (Fig. S1A). Following this recovery period, the pretreated cells showed elevated expression of XBP1, BIP, HMOX1, and GADD34, indicating sustained activation of the ATF6 and PERK branches of the UPR (Fig. 2H–K and S2J–S2M). In parallel, the RIDD targets *BLOC1S* and *SCARA3* were significantly downregulated, indicating persistent engagement of the IRE1α-RIDD pathway in pretreated cells (Fig. 2L–M and S2N–S2O). In contrast, *XBP1* mRNA splicing remained unchanged in response to genotoxic treatment, whereas positive controls treated with tunicamycin or the sarco/endoplasmic reticulum Ca²⁺-ATPase inhibitor thapsigargin exhibited robust splicing (Fig. 2L–N and S2N–S2O). These findings show that prolonged low-dose cisplatin or doxorubicin pretreatment activates all three arms of the UPR without inducing XBP1 splicing. Sustained engagement of these adaptive pathways appears to be a defining feature of pretreated cells and may underlie the acquisition and maintenance of chemoresistance. (Figs. 1, S1, S2, and S2).

### Tunicamycin induces ERCYS and increases chemoresistance

**Fig. 3:**
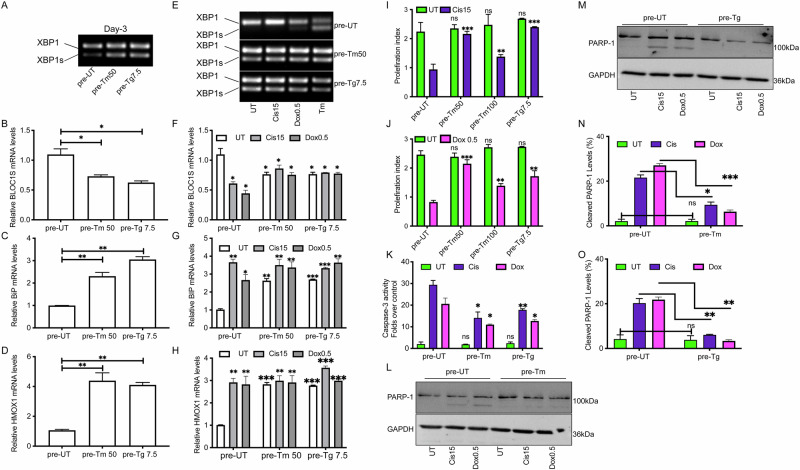
Pre-activation of the UPR confers a prosurvival effect on cells treated with cisplatin and doxorubicin. **A** Representative EtBr-stained agarose gel of XBP1 cDNA amplicons in A549 cells after 3 days of treatment with 50 ng/mL Tunicamycin (Tm) or 7.5 nM Thapsigargin (Tg). N = 3. **B–D** qPCR of relative mRNA levels of BLOC1S, BiP, and HMOX1 in A549 cells treated with 50 ng/mL Tm or 7.5 nM Tg. N = 3. **E** Representative EtBr-stained agarose gel of XBP1 cDNA amplicons in A549 cells pretreated with ER stressors (50 ng/mL Tm or 7.5 nM Tg) for 3 days and then challenged with high concentrations [μMs] of cisplatin and doxorubicin. As a positive control, cells were treated with Tm (250 ng/mL). N = 3. **F–H** qPCR of relative mRNA levels of BLOC1S, BiP, and HMOX1 in A549 cells pretreated with ER stressors (50 ng/mL Tm or 7.5 nM Tg) and then treated with cisplatin and doxorubicin [μMs]. N = 3. **I–J** XTT assay in ER stressors-pretreated cells (50 ng/mL Tm or 7.5 nM Tg) challenged with high concentrations [μMs] of doxorubicin as indicated in A549 cell lines. N = 3. **K** Fold change of Caspase3/7 activity in 50 ng/mL Tm or 7.5 nM Tg pretreated A549 cells and then treated with the indicated concentrations of cisplatin or doxorubicin [μMs]. N = 3. **L**, **M** Representative immunoblots of PARP-1 and GAPDH in A549 cells pretreated with Tm and Tg (50 ng/mL Tm or 7.5 nM Tg) and then treated with cisplatin and doxorubicin [μMs]. **N**, **O** Quantification of cleaved PARP-1 levels as shown in M and N. All experiments were done in biological triplicates. (***p < 0,001, **p < 0,01, *<0.05).

Because cisplatin and doxorubicin pretreatments induced ER stress and UPR activation, we asked whether UPR is a necessary precondition for cancer chemoresistance. Accordingly, we treated A549 and MCF-7 cells with subtoxic levels of Tm and Tg for three days before treatment with high concentrations of cisplatin or doxorubicin. In cells pretreated with Tm or Tg for three days, XBP1 was found mainly in the spliced form, indicating an activation of the IRE1/Xbp1 arm of the UPR (Figs. 3A). BLOC1S mRNA levels decreased, suggesting an activation of the RIDD arm of IRE1 (Figs. 3B and S3A). We then tested the activation of PERK and ATF6 by looking at the levels of HMOX1 and BIP, respectively. Both genes were induced after Tm and Tg pretreatment for three days, indicating an activation of the full UPR arms (Figs. 3C, D and S3B-S3C). Adding cisplatin and doxorubicin at high concentrations did not change the levels of spliced XBP1. Still, this further decreased BLOC1S levels in cells never treated with ER stressors, compared to pretreatment with Tg and Tm (Figs. 3E, F and S3D). Under those conditions, all treatments tested activated PERK and ATF6 pathways similarly to IRE1 (Figs. 3G, H and S3E, S3F).

Despite activation of the UPR, which is a marker of proteostasis perturbation and stress conditions in the ER, proliferation rates during cisplatin and doxorubicin challenges were higher than in their control counterparts (Fig. 3I–J, and Fig. S3G, S3H). Proliferation was inversely correlated with apoptotic activity, as caspase-3/7 activation and PARP-1 cleavage were markedly reduced in Tm- or Tg-pretreated cells exposed to high concentrations of cisplatin or doxorubicin (Fig. 3K). Consistent with this, levels of cleaved PARP-1 were substantially diminished in both Tm- and Tg-pretreated cells (Figs. 3L–O and S3I). These findings support the notion that mild, subtoxic ER stress and consequent UPR activation are sufficient to enhance proliferation and suppress proapoptotic signaling, thereby preconditioning cells for increased survival under genotoxic stress.

Building on these findings, we selectively inhibited IRE1, PERK, and ATF6 using MKC-3946, GSK2606414, and Ceapin-A7, respectively. Each inhibitor effectively suppressed activation of its downstream targets: MKC-3946 prevented tunicamycin-induced downregulation of *BLOC1S1*, GSK2606414 blocked induction of *HMOX1* and *GADD34*, and Ceapin-A7 abolished *BiP* upregulation (Fig. S3J–S3M).

We next assessed the impact of UPR inhibition on cisplatin resistance. In the presence of UPR inhibitors, cisplatin-treated cells displayed increased annexin-V positivity and reduced viability relative to controls, indicating that suppression of UPR signaling prevented the acquisition of chemoresistance (Fig. S3N). To further evaluate the role of ER stress in this process, we pretreated cells with tauroursodeoxycholic acid (TUDCA), a chemical chaperone that alleviates ER stress. TUDCA pretreatment fully abrogated cisplatin-induced chemoresistance and sensitized cells to high doses of cisplatin (Fig. S3N). Consistent results were obtained in clonogenic assays, where both UPR inhibition and ER stress alleviation significantly reduced colony survival compared with controls (Fig. S3O–S3P). Taken together, these results support the conclusion that cisplatin-induced chemoresistance in cancer cells is associated with ER stress and UPR activation, and that suppression of UPR signaling or alleviation of ER stress reduces this response.

**Fig. 4:**
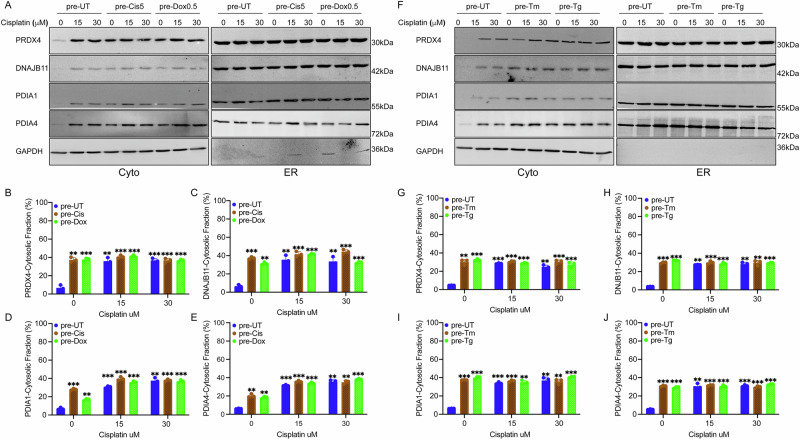
ER to cytosol signaling (ERCYS) is active in chemoresistant cells and cells pre-treated with ER stress inducers. **A** Subcellular protein fractionation (Digitonin fraction) of PRDX4, DNAJB11, PDIA1, and PDIA4 in A549 cells pretreated with cisplatin and doxorubicin and then treated with high concentrations [μMs]. of cisplatin and doxorubicin. N = 3. **B–E** Quantification of the refluxed ER proteins PRDX4, DNAJB11, PDIA1, and PDIA4 as in A, respectively. **F** Subcellular protein fractionation (Digitonin fraction) of PRDX4, DNAJB11, PDIA1, and PDIA4 in A549 cells pretreated with ER stressors 50 ng/mL Tm or 7.5 nM Tg and then treated with high concentrations [μMs] of cisplatin and doxorubicin. N = 3. **G–J** Quantification of the refluxed ER proteins PRDX4, DNAJB11, PDIA1, and PDIA4 as in F respectively. All experiments were done in biological triplicates. (***p < 0,001, **p < 0,01, *<0.05).

Furthermore, we previously showed that ER stress activates the reflux of proteins from the ER to the cytosol by activating a pro-survival mechanism called ER to cytosol signaling (ERCYS) [24–26, 38]. Here, we tested whether ER stress induction in chemoresistant cells is enough to stimulate ERCYS. Hence, we analyzed the localization of the endogenous lumenal proteins peroxiredoxin-4 (PRDX4), DnaJ Heat Shock Protein Family (Hsp40) Member B11 (DNAJB11), protein disulfide isomerase-1 (PDIA1), and protein disulfide isomerase-4 (PDIA4) in pretreated cells compared to control cells. In the control cells, the tested ER proteins were mainly localized to the ER. In contrast, cells pretreated with low cisplatin and doxorubicin showed higher levels of the proteins refluxed to the cytosolic fraction (Fig. 4A–E). Treating cells with high concentrations of cisplatin and doxorubicin resulted in the reflux of the tested proteins even in pre-UT conditions (Fig. 4A–E). Although the pretreated cells accumulated higher levels of cytosolically localized ER proteins, cisplatin at high concentrations resulted in equal levels in all treatments, indicating that the maximum reflux levels were reached.

In addition, cells pretreated with low and subtoxic concentrations of Tm and Tg for three days showed increased cytosolic localization of PRDX4, DNAJB11, PDIA1, and PDIA4 (Fig. 4F–J). The levels of the cytosolically localized ER proteins in cells pretreated with Tm or Tg were equal to those in cells pretreated with cisplatin and doxorubicin (Fig. 4).

These results indicate that ERCYS is constitutively active in chemoresistant cells and in cells pretreated with ER stress inducers. Although high-dose drug treatment equalizes cytosolic ER protein levels across conditions, pre-activation of ERCYS in pretreated cells may provide a protective advantage over post-activation (Figs. 3, S3, and 4).

### Pre-activated ERCYS inhibits proapoptotic pathways in the cytosol

Because wt-p53 induction in response to cisplatin and doxorubicin was partially inhibited in chemoresistant cells (Fig. 1K), and given that ERCYS activation has been linked to non-genetic inhibition of wt-p53, we tested whether ERCYS pre-activation contributes to the suppression of wt-p53 transcriptional activity (Fig. S1). To this end, A549 cells were transfected with a luciferase reporter under the control of a p53 DNA-binding site and assayed under different conditions. Pretreatment with Tm or Tg for three days did not alter basal wt-p53 activity. As expected, high-dose cisplatin significantly increased reporter activity in control cells; however, this induction was markedly attenuated in Tm- or Tg-pretreated cells with active ERCYS (Fig. S4A).

Interestingly, cells pretreated with low-dose cisplatin displayed higher wt-p53 activity than untreated controls (Fig. S4B). While high-dose cisplatin or doxorubicin strongly induced wt-p53 in pre-UT cells, only a modest increase was observed in cisplatin-pretreated cells (Fig. S4B). These findings suggest that during the adaptation phase to low-level stressors (Tm, Tg, or chemotherapy), cells adopt distinct mechanisms to enhance fitness. Specifically, mild ER stress perturbs proteostasis, activating ERCYS and driving ER protein reflux into the cytosol. This raises the question of whether refluxed proteins directly interact with key apoptotic regulators such as caspase-3 or transcription factors like wt-p53 to suppress their activity (Fig. S4).

### Expelled PDIA4 interacts with Caspase-3 and p53

**Fig. 5:**
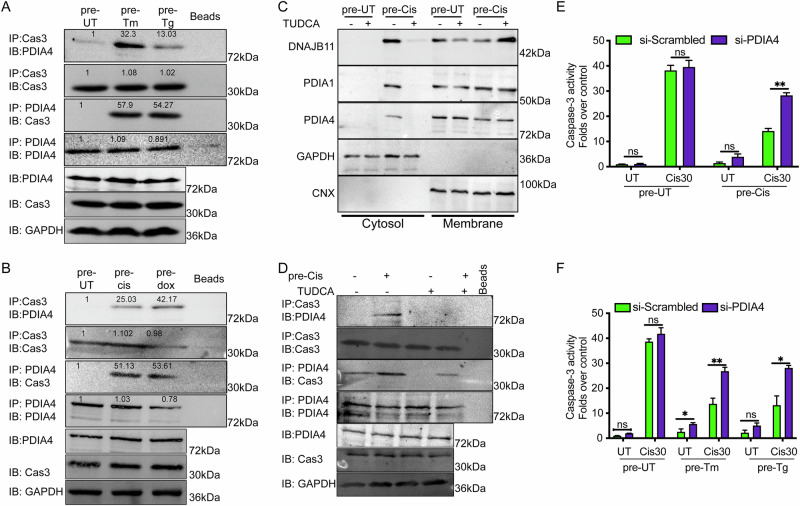
PDIA4 interacts with caspase3 and inhibits its activity. **A** Representative immunoblot showing the interaction between Caspase-3 and PDIA4 in A549 cells treated with tunicamycin (Tm 50 ng/mL) or thapsigargin (Tg 7.5 nM) for 3 days. (Relative band intensities in white). **B** Representative immunoblot showing the interaction between Caspase-3 and PDIA4 in A549 cells treated with 5 μM Cisplatin or 0.05 μM doxorubicin for 14 days. (Relative band intensities in white). **C** Subcellular protein fractionation (Digitonin fraction) of DNAJB11, PDIA1, and PDIA4 in A549 cells co-pretreated with 5 μM cisplatin and Tauroursodeoxycholic acid (TUDCA), N = 3. **D** Representative immunoblot showing the interaction between Caspase-3 and PDIA4 in A549 cells co-pretreated with 5 μM cisplatin and TUDCA. Folds changes of Caspase3/7 activity after treatment with the indicated concentrations of cisplatin (cis) or doxorubicin (dox) in A549 cells pretreated with 5 μM cisplatin (**E**) or ER stressors 50 ng/mL Tm or 7.5 nM Tg (**F**). N = 3. All experiments were done in biological triplicates. (***p < 0,001, **p < 0,01, *<0.05).

PDI proteins were recently shown to negatively regulate procaspase-3/7 activity under various conditions [39–41]. In addition, elevated PDIA4 expression has been associated with poor prognosis in several cancer types, including glioma, renal cell carcinoma, cervical cancer, and others [42–45]. Hence, we tested whether the expelled PDIA4 forms an inhibitory interaction with caspase-3 and wt-p53 - two highly attenuated pathways in chemoresistant cells (Figs. 1, 3, S3, and S4). In normal conditions, no interaction occurs between PDIA4 and caspase-3 that localize into different cellular compartments (Figs. 4 and 5A). Treating cells with low doses of Tm or Tg for three days significantly increased the interaction between PDIA4 and Caspase-3 (Fig. 5A). Moreover, in conditions where cells were pretreated with cisplatin or doxorubicin for two weeks, PDIA4-caspase3 interaction was very robust (Fig. 5B). To assess whether ER proteostasis perturbation is required, we treated cells with low-dose cisplatin in the presence of the chemical chaperone TUDCA. Cotreatment with TUDCA reduced the cytosolic abundance of ER proteins (Fig. 5C), indicating that ER stress is required for ERCYS activation. Consistently, TUDCA reversed the cisplatin-induced PDIA4–caspase-3 interaction (Fig. 5D). Together, these findings suggest that ER stress promotes aberrant PDIA4–caspase-3 interaction in chemoresistant cells, which may contribute to apoptosis resistance.

To determine whether the PDIA4–caspase-3 interaction occurs in the cytosol, we performed co-immunoprecipitation of PDIA4 from digitonin-permeabilized fractions and assessed its association with caspase-3. Following tunicamycin pretreatment, PDIA4 was detected in the cytosol and co-precipitated with caspase-3 (Fig. S5A–B), confirming that the interaction occurs after ER-to-cytosol translocation. Consistently, a PDIA4 variant lacking the signal peptide and ER-retention sequence did not interact with caspase-3, whereas ER-localized PDIA4 showed robust interaction under tunicamycin stress (Fig. S5C–D). In this setting, caspase-3 activity was markedly suppressed in cells overexpressing ER-localized PDIA4 compared to cells expressing the cytosolic variant (Fig. S5E), indicating that PDIA4 must originate from the ER to bind caspase-3. This requirement likely reflects the need for proper folding or post-translational modifications acquired within the ER. Supporting this, glycosylated PDIA4 was found to interact with cytosolic caspase-3, demonstrating that PDIA4 first enters the ER, undergoes glycosylation, and subsequently refluxes to engage caspase-3 (Fig. 5F–H).

Immunofluorescence further confirmed the cytosolic localization of the PDIA4–caspase-3 complex (Fig. S5I). Together, these findings establish that ER stress promotes PDIA4 reflux into the cytosol, where it directly interacts with caspase-3, thereby mechanistically linking ERCYS to the suppression of apoptotic signaling.

To investigate the nature of this interaction, we silenced PDIA4 and assayed the activity of caspase-3 in cells pretreated with ER stress inducers or anti-cancer drugs. The cells treated with low doses of cisplatin for two weeks demonstrated higher caspase-3 activity than the controls. The addition of high levels of cisplatin after two weeks markedly increased caspase activity in the scrambled and PDIA4-silenced pre-UT cells. In pre-Cis cells (chemoresistant cells), the increase in caspase-3/7 activity was significantly higher in the PDIA4-silenced cells compared to the scrambled cells (Fig. 5E and Fig. S6A).

Moreover, silencing PDIA4 resulted in the rescue of caspase-3/7 activity in cells pretreated with Tm and Tg for 3 days and later challenged with cisplatin (Fig. 5F). The rescue of caspase-3 activity after silencing PDIA4 indicates an inhibitory interaction. These results further suggest that in the pretreated cells with either cisplatin, Tm, or Tg, PDIA4 is refluxed to the cytosol to bind and inhibit caspase-3. Moreover, silencing PDIA4 rescues caspase-3 activity, indicating that it is the primary inhibitor of caspase-3/7 activity in the cytosolic fraction.

**Fig. 6:**
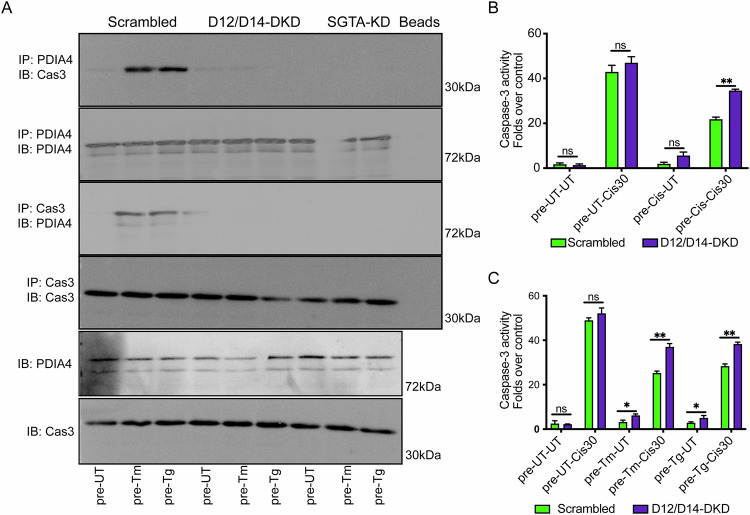
DNAJB12/14 and SGTA are needed to reflux PDIA4 and caspase3/7 inhibition. **A** Representative immunoblot showing the interaction between caspase-3 and PDIA4 in A549 cells treated with tunicamycin (Tm 50 ng/mL) and thapsigargin (Tg 7.5 nM) for 3 days in control cells and in DNAJB12/14-depleted or SGTA-depleted cells. N = 3. Folds changes of Caspase3/7 activity after treatment with the 30 μM cisplatin (cis) in DNAJB12/14-depleted A549 cells pretreated with 5 μM cisplatin (**B**) or ER stressors (**C**). N = 3. All experiments were done in biological triplicates. (***p < 0,001, **p < 0,01, *<0.05).

We found that PDIA4 inhibits cytosolic caspase-3 activity during ER stress in chemoresistant cells. We therefore hypothesized that this effect depends on PDIA4 refluxed from the ER rather than on a mislocalized cytosolic pool. To test this, we silenced DNAJB12 and DNAJB14, as well as SGTA. In scrambled controls, PDIA4 bound caspase-3 in cells pretreated with tunicamycin or thapsigargin compared with untreated cells (Fig. 6A). Silencing DNAJB12/14 abolished this interaction under all conditions (Figs. 6A, Fig. S6B). Similarly, SGTA knockdown phenocopied the double knockdown (Figs. 6A, S6C–D). This loss of interaction resulted from impaired reflux of PDIA4 to the cytosol in SGTA- or DNAJB12/14-silenced cells (Fig. S6E–H).

To further dissect this pathway, we examined the interaction of refluxed PDIA4 and PDI with caspase-3 and DNAJB12/14. In scrambled cells and SGTA-silenced cells, refluxed proteins associated with DNAJB12 and DNAJB14; however, in the absence of SGTA, they failed to reach the cytosol despite maintaining their interaction with DNAJB12/14 (Fig. S6I). Additionally, SGTA silencing prevented the PDIA4–caspase-3 interaction, indicating that SGTA is required for PDIA4 redistribution to the cytosol and the subsequent formation of this inhibitory complex (Fig. S6I). These results suggest that engagement with DNAJB12/14 constitutes an initial step, followed by transfer to cytosolic chaperones and cochaperones before PDIA4 interacts with new cytosolic protein partners (Fig. S6I).

Consistently, caspase-3 activity was significantly higher in double knockdown cells compared with controls (Fig. 6B, C). Moreover, the reduction of caspase-3/7 activity by Tm, Tg, or cisplatin pretreatment was attenuated when PDIA4 was silenced, confirming that cytosolic reflux of PDIA4 is essential for caspase-3/7 activity (Fig. 5). Together, these findings establish that DNAJB12, DNAJB14, and SGTA are required for PDIA4 reflux to the cytosol, where it binds caspase-3 and promotes cancer cell resistance.

We next examined whether PDIA4 contributes to the suppression of wild-type p53 (wt-p53) activity. Cisplatin or doxorubicin treatment increased the association between PDIA4 and wt-p53, and this interaction was further enhanced when cells were pretreated with Tm or Tg for three days before genotoxic stress (Fig. S6J). To assess the functional impact, PDIA4 was silenced in A549 cells, followed by Tm or Tg pretreatment and cisplatin exposure. In cells treated with cisplatin alone, wt-p53 activity was elevated, whereas Tm pretreatment reduced wt-p53 activity in control cells (Fig. S6K). Under these conditions, PDIA4 knockdown partially restored wt-p53 transcriptional activity, indicating that PDIA4 contributes to wt-p53 inhibition (Fig. S6K). Given that other ER-resident proteins, such as AGR2, also interact with and inhibit wt-p53 [26], full restoration was not achieved.

To further dissect this mechanism, DNAJB12, DNAJB14, or SGTA knockdown blocked the cytosolic translocation of PDIA4 and other ER proteins (Fig. S6) and significantly restored wt-p53 levels (Fig. S6K–S6L). Together, these findings indicate that DNAJB12, DNAJB14, and SGTA mediate the reflux of PDIA4 and likely a broader set of ER-resident proteins into the cytosol, where they suppress wt-p53 activity and contribute to its inhibition in chemoresistant cells.

**Fig. 7:**
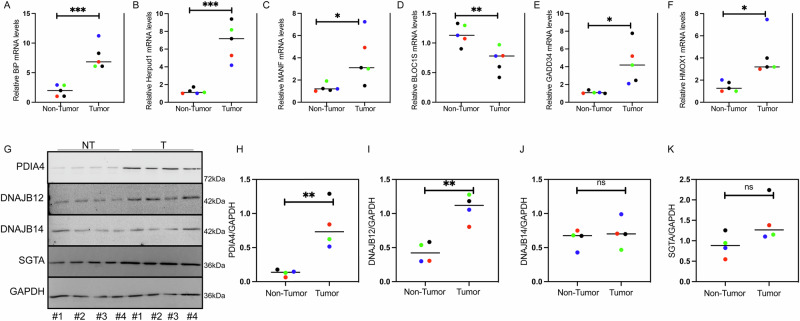
PDIA4 and DNAJB12 are highly expressed in colorectal cancer (CRC) and are associated with the induction of the UPR. **A**, **B** qPCR of relative mRNA levels of the ATF6-regulated genes BIP and HERPUD1 in CRC patients. **C**, **D** qPCR of relative mRNA levels of the IRE1-regulated genes MANF and BLOC1S in CRC patients. **E**, **F** qPCR of relative mRNA levels of the PERK-regulated genes GADD34 and HMOX1 in CRC patients. **G** Representative western blot of PDIA4, DNAJB12, DNAJB14, SGTA, and GAPDH in samples from colorectal cancer patients. **H–K** Quantification of PDIA4, DNAJB12, DNAJB14, and SGTA as shown in G. (***p < 0,001, **p < 0,01, *<0.05).

Finally, to correlate these findings with clinical outcomes, we performed qPCR on malignant and matched normal tissues from colorectal cancer patients. IP, HERPUD1, GADD34, HMOX1, and MANF mRNA levels were significantly upregulated in tumor samples, indicating robust activation of all three arms of the UPR (Fig. 7A–E). Moreover, BLOC1s levels are downregulated in the tumors compared to normal tissue, indicating a full activation of IRE1, including the RIDD activity (Fig. 7D). In the tumor tissues, the protein levels of PDIA4 and DNAJB12 were also higher than normal tissues (Fig. 7G–K). Those data indicate that in malignant tissues, the levels of DNAJB12 and PDIA4 may play adaptive and pro-cancerous functions to increase cancer cell fitness by refluxing PDIA4 from the ER to the cytosol to non-genetically inhibit caspase-3/7 activity. Moreover, using the TCGA survival analysis database [46, 47] we found that high copy number of PDIA4 is associated with poor prognosis in many cancer types, including, Uterine Corpus Endometrial Carcinoma (UCEC)Thyroid carcinoma (THCA), Low grade glioma (LGG), mesothelioma (MESO),Prostate adenocarcinoma (PRAD) (Fig. S7A–E).

## Discussion

Here, we describe a process by which cancer cells gain chemoresistance to different chemotherapeutic agents, relying on the perturbation of proteostasis in the endoplasmic reticulum and activation of a pro-survival mechanisms including the UPR and ERCYS. These cells increase proliferation by inhibiting the caspase-3/7 pathway. Moreover, the data presented here show that cells that gain resistance to cisplatin are also resistant to doxorubicin and vice versa. This cross-resistance is also associated with lower activity of caspase-3/7 and inhibition of PARP-1 cleavage, suggesting that upstream pathways activated in the process may act as inhibitors of caspase-3.

ER stress and UPR activation can serve adaptive functions in cancer cells, promoting tumor progression and contributing to the acquisition of chemoresistance, and are therefore considered promising therapeutic targets [12, 13]. In this study, we demonstrate that resistant cells maintained UPR activation following prolonged exposure to low concentrations of cisplatin or doxorubicin, reinforcing the link between resistance and this adaptive process. Importantly, UPR activation was not simply a consequence of chemoresistance but was sufficient to promote it: subtoxic ER stress induction enhanced cancer cell survival and proliferation during cytotoxic challenge, as observed in cells pretreated with low-dose cisplatin or doxorubicin. Cell viability assays further revealed that this ER stress–dependent chemoresistance requires the coordinated activity of all three UPR branches, since inhibition of IRE1, ATF6, or PERK impaired resistance and increased cisplatin-induced cytotoxicity. Together, these findings identify persistent and integrated UPR signaling as a key mechanism by which cancer cells acquire chemoresistance, highlighting it as a tractable therapeutic vulnerability.

Mechanistically, ERCYS emerged as a critical determinant of chemoresistance. We previously reported that ERCYS mediates the reflux of ER-resident proteins into the cytosol in response to ER stress, a process associated with non-genetic inhibition of wt-p53 and considered an adaptive, pro-cancerous mechanism [23, 24, 26]. In the present study, we demonstrate that ERCYS is engaged in chemoresistant cells, where it selectively targets pro-apoptotic pathways. Notably, members of the PDI family were detected in the cytosol of chemoresistant cells even before exposure to chemotherapeutic drugs, indicating that ERCYS is active at baseline. Among these, PDIA4 emerged as a xkey substrate: once refluxed, cytosolic PDIA4 acquired novel functions, binding to and inhibiting two central proapoptotic regulators, wt-p53 and caspase-3/7. This inhibitory activity depended on the ER membrane–anchored proteins DNAJB12 and DNAJB14, which regulate ERCYS, as well as the cytosolic co-chaperone SGTA. Silencing SGTA disrupted PDIA4 reflux, blocked its interaction with caspase-3/7, and restored the activity of both wt-p53 and caspase-3/7. Collectively, these findings position PDIA4 as a critical effector of ERCYS and highlight ERCYS as a central mechanism by which cancer cells neutralize apoptosis and acquire chemoresistance.

Pre-activation of ERCYS in chemoresistant cells functions as an adaptive mechanism that suppresses caspase-3 before exposure to high doses of cisplatin, thereby conferring an enhanced survival advantage. In contrast, in pre-UT cells, ERCYS activation coincides with caspase-3 induction (Fig. 4), resulting in less effective protection. This highlights the importance of timing: constitutive or pre-emptive inhibition of caspase-3 is more protective than attempting to block pro-apoptotic signaling once it has already been triggered. Supporting this model, treatment with TUDCA alleviated ER stress, inhibited ERCYS, and abrogated chemoresistance. Mechanistically, TUDCA prevented PDIA4–caspase-3 interaction (Fig. 5C–D) and reduced ERCYS-dependent survival, consistent with a role for PDIA4 reflux in mediating resistance. Together, these data indicate that chemoresistant cells exploit ERCYS pre-activation to preempt apoptotic signaling, whereas pharmacological inhibition of ER stress with TUDCA, or genetic inhibition of ERCYS via DNAJB12/14 and SGTA effectively blocks protein reflux and restores caspase-3–dependent cell death.

Furthermore, we examined whether PDIA4 requires ER processing to engage caspase-3. A cytosolic PDIA4 variant lacking both the signal peptide and the ER-retention sequence neither interacted with caspase-3 nor significantly altered its activity, despite being abundantly expressed in the cytosol. By contrast, ER-targeted PDIA4 strongly suppressed caspase-3 activity and efficiently co-immunoprecipitated with the caspase-3 under ER stress (Fig. S5C–E). Furthermore, a glycosylated form of PDIA4 was detected interacting with caspase-3 in the digitonin fraction, indicating that this variant originated from the ER before refluxing into the cytosol (Fig. F-H). Together, these results suggest that ER-originated PDIA4 undergoes specific maturation steps that enable its cytosolic function as a regulator of caspase-3 activity. Importantly, this mechanistic requirement underscores how ERCYS-mediated reflux of ER-processed PDIA4 provides chemoresistant cells with a strategy to disable apoptosis, thereby reinforcing the role of ERCYS as a central driver of stress-adaptive survival.

The clinical significance of this mechanism is underscored by genomic data. Analysis of the TCGA dataset revealed that high PDIA4 copy number is associated with poor prognosis and reduced patient survival (Fig. S7A–E) [46]. Together those data may indicate that high levels of PDIA4 in cancer patients may work by inhibiting caspase3 and p53 in the cytosol of those patients.

In summary, cancer cells acquire chemoresistance by pre-activating the ER stress–dependent pro-survival mechanism ERCYS, which mediates cytosolic reflux of select ER-resident proteins, including PDIA4. Cytosolic PDIA4 inhibits key pro-apoptotic regulators, wt-p53 and caspase-3/7, in a process dependent on DNAJB12, DNAJB14, and SGTA, allowing cells to survive chemotherapy and develop cross-resistance. ERCYS represents an adaptive, reversible mechanism by which cancer cells remodel interorganellar proteostasis to evade apoptosis through non-genetic reprogramming of protein localization and function. Targeting ERCYS or its components—PDIA4, DNAJB12, DNAJB14, and SGTA—restores apoptotic signaling and sensitizes cells to chemotherapy. Combining pharmacological ERCYS inhibition with conventional low-dose chemotherapy may enhance efficacy while reducing toxicity, offering a promising strategy to overcome drug resistance in the clinic.

## Supplementary information

SUPPLEMENTAL File

SUPPLEMENTAL Figure S1

SUPPLEMENTAL Figure S2

SUPPLEMENTAL Figure S3

SUPPLEMENTAL Figure S4

SUPPLEMENTAL Figure S5

SUPPLEMENTAL Figure S6

SUPPLEMENTAL Figure S7

## Data Availability

Raw and processed data from this study are available from the lead contact upon request. Additional information needed to reanalyze the data can also be obtained from the lead contact. All data generated or analyzed in this work are provided within the article and its supplementary files.
