# Correction: Non-genetic inactivation of caspase-3 and P53 increases cancer cell fitness by PDIA4 redistribution

**DOI:** 10.1038/s41388-025-03616-5

**Published:** 2025-10-30

**Authors:** Gal Twito, Faiza Amterat Abu Abayed, Ayelet Gilad, Suma Biadsy, Noa Gavriel, Suad Sheikh Suliman, Yarden Mizrahi, Hila Megged, Mor Tenenboim, Naim Abu-Freha, Aeid Igbaria

**Affiliations:** 1 Department of Life Sciences, Ben-Gurion University of the Negev, Beer Sheva, Israel; 2 Institute of Gastroenterology and Liver Diseases, Soroka Medical Center, Faculty of Health Sciences, Ben Gurion University of the Negev, Beer Sheva, Israel

**Keywords:** Apoptosis, Stress signalling

Correction to: *Oncogene* 10.1038/s41388-025-03606-7, published online 21 October 2025

In this article, the title was incorrectly given as ‘On-genetic inactivation of caspase-3 and P53 increases cancer cell fitness by PDIA4 redistribution’ but should have been ‘Non-genetic inactivation of caspase-3 and P53 increases cancer cell fitness by PDIA4 redistribution’.

The original article has been corrected.

