# Supplementary material for: Non-genetic inactivation of caspase-3 and P53 increases cancer cell fitness by PDIA4 redistribution
Source: Oncogene. 2025 Oct 21;44(47):4565–75. doi: 10.1038/s41388-025-03606-7 (PMC12623246; doi:10.1038/s41388-025-03606-7)
Supplement: Supplementary file 1 — SUPPLEMENTAL File [file 41388_2025_3606_MOESM1_ESM.pdf]

SUPPLEMENTARY MATERIALS

Non-genetic Inactivation of Caspase-3 and P53 Increases Cancer Cell Fitness by PDIA4 Redistribution

Gal Twito[1], Faiza Amterat Abu Abayed[1], Ayelet Gilad[1], Suma Biadsy[1], Noa Gavriel[1], Suad Sheikh Suliman[1], Yarden Mizrahi[1], Hila Megged[1], Mor Tenenboim[1], Naim Abu-Freha[2], , Aeid Igbaria[1#]

[1]Department of Life Sciences, Ben-Gurion University of the Negev, Beer Sheva, Israel

[2]Institute of Gastroenterology and Liver Diseases, Soroka Medical Center, Faculty of Health Sciences, Ben Gurion University of the Negev, Beer Sheva, Israel

[#]Correspondence: aigbaria@bgu.ac.il

Supplementary Materials and Methods:

Colony formation assay: Five hundred cells were seeded in 6-well plates and treated with the indicated compounds for the specified duration, beginning 24 h after plating. Colonies were fixed with a methanol:acetic acid solution (5:1) at room temperature for 20 min, then stained with GelCode™ Blue Safe Protein Stain (Thermo Fisher, Cat. No. 24594) in H_2O for 2 h. Colony numbers were quantified using ImageJ software.

Annexin V / PI Apoptosis Assay: Cell death was assessed using the Annexin V/PI Apoptosis Assay, following the manufacturer's instructions (PE Annexin V Apoptosis Detection Kit, BD Pharmingen, Cat. 559763). Briefly, 1×10^5 cells/ml were resuspended in 200 µl binding buffer, and 1 µl Annexin V was added. After a 30-min incubation at room temperature, cells were centrifuged, washed with 500 µl binding buffer, and resuspended in 200 µl fresh buffer containing 5 µl propidium iodide. Samples were analyzed on an iCyt sy3200 Benchtop Cell Sorter/Analyzer (Sony Biotechnology, San Jose, CA, USA). A minimum of 10,000 events were acquired, displayed on dot plots, and analyzed using EC800 software (Sony Biotechnology).

Generation of PDIA4 Variants: Cytosolic PDIA4 was generated by PCR cloning of the PDIA4 open reading frame (ORF) lacking both the ER signal peptide and the C-terminal KDEL sequence. To generate glycosylated PDIA4, a glycosylation site was introduced on a linker positioned immediately upstream of the KDEL sequence. The primers used in this study were as follows:

ER-PDIA4:

Forward:5′CGCCGGATCCACCATGAGGCCCCGGAAAGCCTTCCTGCTCC3′
Reverse:5′CAGACGCGGCCGCTTCAAAGCTCTTCCTTCTTATCGTCGTCATCCTTGTA
ATCGGTCCTGCTCAGTTTTGTGGCATGTTC3′.
Cytosolic PDIA4:
Forward:5′CGCCGGATCCACCATGGCAAAGGTCTCCTCGCTCATTGAGAG-3′
Reverse:5′GAGGCGGCCGCATTATTACTTATCGTCGTCATCCTTGTAATCCTTTTTCCC
TAGTCTTTCATGGTCTC-3′.
N-Glyc-PDIA4:
Forward:5′CTCCGCGGATCCTACCATGAGGCCCCGGAAAGCCTTCCTG-3′.
Reverse:5′TCAGGGCGGCCGCCCTTCAAAGCTCTTCCTTCTTGTCGTCATCGTCTTT
GTAGTCAGTACCAGTAACATTTGTGGTCCTGCTCAGTTTTGTGGCATGTTC-3′

Knockdown Experiments: Small-interfering RNAs (siRNAs) targeting PDIA4 were purchased from Santa Cruz Biotechnology (Catalog # sc-44571). For CRISPR interference (CRISPRi)-mediated knockdown of SGTA, DNAJB12, and DNAJB14, experiments were performed following the protocol described by [1] The specific oligonucleotides used in this study are listed in Supplementary Table-S1.

Supplementary Tble-S1

SGTA	DNAJB12	DNAJB14
gaccgatccccgacccaccga<br>ggtctgcggctcggggccca<br>gtctggggtctgcggctcg<br>gaaggaagtgacgcaacgtag<br>ggcgcctttcttttgcgc<br>gccagggtcaccgcgacccgc<br>gcctactcacaggaccccgc<br>gcgcaagcgcaaccgtcgg<br>gcacaggcgcgttaatga<br>gtaagagtttggggatcgtg	gctcaaaactcgggaaaggag<br>ggatgtcatcactccgcgcc<br>gcacgcccagattccatgttc<br>gtcttctaagaaagggacggc<br>gtccctttcttagaagacata<br>gcgaagggctgcgccacccgc<br>ggcgcagcccttcgctcgcc<br>gctggtgtaggtgtcaataa<br>gcccggctgcccgcgacgcgc<br>gacctccggaacatggaatc	ggcggggaggccgccgtgagg<br>gaggtctcaccgcgggccg<br>ggctgcgacggcccgggcgga<br>gctgcgaggcccgcccgtccgcc<br>gcctcgcagctccgcctcctca<br>ggaagaaggagcaagctatgg<br>gctgccgccgcacgcccccagc<br>gagaaatgtgtcgagatcgcc<br>gaagggcggaagccgccgccg<br>gcggcagcggggaggagcgca

Supplementary Table-S1: list of oligos used to knockdown SGTA, DNAJB12, and DNAJB14 as described by [1]

Supplement Table-S2

**Antibody (Host)**	**Company/Cat. No.**
DNAJB12 (Rabbit)	Ptg/16780
DNAJB14 (Rabbit)	Ptg/16501
SGTA (Rabbit)	Ptg/11019
SGTA (Mouse)	Ptg/ 60305-1-Ig
PDIA1 (Rabbit)	Ptg/11245-1-AP
PDIA1 (Mouse)	Ptg/66422-1-Ig
PDIA4 (Rabbit)	Ptg/14712-1-AP
PDIA4 (Mouse)	Ptg/66365-1-Ig
AGR2 (Rabbit)	Ptg/12275-1-AP
AGR2 (Mouse)	SantaCruz/sc-101211
AGR2 (Rat)	Biolegend/943102
DNAJB11 (Rabbit)	Ptg/15484-1-AP
PRDX4 (Rabbit)	Ptg/10703-1-AP
PRDX4 (Mouse)	Ptg/ 60286-1-Ig
pan-p53 (DO-1) (Mouse)	SantaCruz/sc-126
p53 (Rabbit)	Ptg/ 10442-1-AP
p53 (Rabbit)	Ptg/60283
phospho-p53 (Ser15) (Rabbit)	Ptg/28961
phospho-p53 (Ser15) (Mouse)	Ptg/ 67826-1-Ig
Caspase-3 (Rabbit)	Cell signaling #9662
Caspase-3 (Mouse)	Ptg/66470-2-Ig
FLAG-DYKDDDDK (Mouse)	Ptg/66008-4
FLAG-DYKDDDDK (Rabbit)	Cell signaling #14793
GADPH (G-9) (Mouse)	SantaCruz/sc-365062
Goat anti-Mouse IgG (H+L) Highly Cross-Adsorbed Secondary Antibody, Alexa Fluor™ Plus 488	Invitrogen/ #A32723
Goat anti-Rabbit IgG (H+L) Highly Cross-Adsorbed Secondary Antibody, Alexa Fluor™ Plus 488	Invitrogen/ #A32731
Goat anti-Rabbit IgG (H+L) Highly Cross-Adsorbed Secondary Antibody, Alexa Fluor™ Plus 647	Invitrogen/ #A32733
Goat anti-Mouse IgG (H+L) Highly Cross-Adsorbed Secondary Antibody, Alexa Fluor™ Plus 647	Invitrogen/ #A32728
Goat anti-Rabbit IgG (H+L) Highly Cross-Adsorbed Secondary Antibody, Alexa Fluor™ Plus 555	Invitrogen/ #A32732
Goat anti-Rat IgG (H+L) Cross-Adsorbed Secondary Antibody, Alexa Fluor™ 488	Invitrogen/ #A-11006
Goat anti-Rat IgG (H+L) Highly Cross-Adsorbed Secondary Antibody, Alexa Fluor™ Plus 647	Invitrogen/ #A48265

Supplementary Table-S2: list of antibodies used in this study.

Supplementary Figure Legends:

Figure S1: (A) Schematic of the experimental workflow used to generate chemoresistant A549 cells and the downstream assays performed. **(B)** Colony formation assay of A549 cells pretreated with cisplatin or doxorubicin for 14 days, followed by challenge with 30 μM cisplatin or 0.5 μM doxorubicin. **(C)** Quantification of the colony formation assay shown in (B). Data represent biological triplicates. Statistical significance: ***$p < 0.001$, **$p < 0.01$, *$p < 0.05$.

Figure S2: (A-B) qPCR of relative mRNA levels of the IRE1-regulated genes BLOC1S and SCARA3 in MCF-7 cells treated with different concentrations [μMs] of cisplatin (Cis) or doxorubicin (Dox) for 14 days N=3. **(C)** qPCR of relative mRNA levels of the IRE1-regulated genes BLOC1S and SCARA3 in MCF-7 cells cotreated with 5μM cisplatin (Cis) and the IRE1 inhibitor MKC-3946. **(D-E)** qPCR of relative mRNA levels of the PERK-regulated genes GADD34 and HMOX1 in MCF-7 cells treated with different concentrations [μMs] of cisplatin (Cis) or doxorubicin (Dox) for 14 days N=3. **(F)** qPCR of relative mRNA levels of the PERK-regulated genes GADD34 and HMOX1 in MCF-7 cells cotreated with 5μM cisplatin (Cis) and the PERK inhibitor GSK2606414. **(G-H)** qPCR of relative mRNA levels of the ATF6-regulated genes BIP and total XBP1 in MCF-7 cells treated with different concentrations [μMs] of cisplatin (Cis) or doxorubicin (Dox) for 14 days N=3. **(I)** qPCR of relative mRNA levels of the ATF6-regulated genes BIP and XBP1 in MCF-7 cells cotreated with 5μM cisplatin (Cis) and the ATF6 inhibitor Ceapin-A7.

(J-K) qPCR of relative mRNA levels of the ATF6-regulated genes BIP and total XBP1 in MCF-7 cells treated with different concentrations [μMs] of cisplatin after pretreatment

with either cisplatin or doxorubicin. N=3. **(L-M)** qPCR of relative mRNA levels of the PERK-regulated genes GADD34 and total HMOX1 in MCF-7 cells treated with different concentrations [μMs] of cisplatin after pretreatment with either cisplatin or doxorubicin. N=3. **(N-O)** qPCR of relative mRNA levels of the IRE1-regulated genes BLOC1S and total SCARA3 in MCF-7 cells treated with different concentrations [μMs] of cisplatin after pretreatment with either cisplatin or doxorubicin. Data represent biological triplicates. Statistical significance: ***p < 0.001, **p < 0.01, *p < 0.05.

<u>Figure S3:</u> **(A-C)** qPCR of relative mRNA levels of BLOC1S, BiP, and HMOX1 in MCF-7 cells treated with 50ng/mL Tm or 7.5nM Tg. N=3. **(D-F)** qPCR of relative mRNA levels of BLOC1S, BiP, and HMOX1 in MCF-7 cells pretreated with ER stressors and then treated with 30 μM cisplatin or 0.5 μM doxorubicin. N=3. **(G-H)** XTT assay in ER stressors-pretreated cells challenged with high concentrations [μMs] of cisplatin and doxorubicin as indicated in MCF-7 cell lines. **(I)** Representative immunoblot of PARP-1 and GAPDH in A549 cells treated with 50ng/mL Tm or 7.5nM Tg for 3 days. N=3. **(J)** qPCR of relative mRNA levels of BLOC1S in cisplatin pretreated cells and the Ire1 inhibitor MKC3946 (5μM). **(K-L)** qPCR of relative mRNA levels of HMOX1 and GADD34 in cisplatin pretreated cells and the PERK inhibitor GSK2606414 (0.5μM). **(M)** qPCR of relative mRNA levels of BIP in cisplatin pretreated cells and the ATF6 inhibitor Ceapin-A7 (5μM). **(N)** Cell viability assay as assessed using the Annexin V/PI Apoptosis Assay in cell cotreated with cisplatin and MKC-3496, GSK2606414, Ceapin-A7, or TUDCA. **(O)** Colony formation assay of A549 cells pretreated with 5μM cisplatin or 0.05μM doxorubicin for 14 days, followed by challenge with 30 μM in the presence of the UPR inhibitors as indicated.

(P) Quantification of the colony formation assay shown in (P). Data represent biological triplicates. Statistical significance: ***p < 0.001, **p < 0.01, *p < 0.05.

Figure S4: **(A)** A549 cells pretreated with ER stressors (50ng/mL Tm or 7.5nM Tg) were transfected with p53-luciferase construct. Cells were treated with cisplatin or doxorubicin, and luciferase experiments were performed. **(B)** cisplatin (5μM) or doxorubicin (0.05μM) pretreated cells were transfected with p53-luciferase construct. Cells were treated with cisplatin or doxorubicin, and luciferase experiments were performed. Data represent biological triplicates. Statistical significance: ***p < 0.001, **p < 0.01, *p < 0.05.

Figure S5: **(A)** Representative immunoblot showing the interaction between Caspase-3 and PDIA4 in the digitonin fraction of A549 cells pretreated with tunicamycin (Tm, 50 ng/mL) for 3 days before adding 30 μM cisplatin. (Relative band intensities in white). **(B)** Subcellular protein fractionation of PDIA4 in A549 cells pretreated with 50 ng/mL Tm and subsequently treated with high concentrations of cisplatin or doxorubicin (input control for experiment shown in A). **(C)** Subcellular protein fractionation (digitonin fraction) of FLAG-PDIA4 in A549 cells expressing ER- or cytosolic-targeted PDIA4. **(D)** Representative immunoblot showing the interaction between Caspase-3 and FLAG-tagged PDIA4 in A549 cells expressing ER-targeted (e-FLAG-PDIA4) or cytosolic-targeted (c-FLAG-PDIA4) constructs and pretreated with Tm (50 ng/mL) for 3 days. **(E)** Folds change in Caspase3/7 activity in A549 cells expressing the cytosolically or ER targeted PDIA4 and treated with 30μM cisplatin. N=3. **(F)** Immunoblot analysis of the glycosylation state of FLAG-PDIA4-Glyc (engineered PDIA4 glycosylation-site construct) in A549 cells treated

with 100 ng/mL or 250 ng/mL Tm for 12 h. **(G)** Subcellular protein fractionation of FLAG-PDIA4-Glyc in A549 cells pretreated with Tm (50 ng/mL). **(H)** Representative immunoblot showing the interaction between Caspase-3 and FLAG-PDIA4-Glyc in the digitonin fraction of A549 cells pretreated with Tm (50 ng/mL), as shown in (F). **(I)** Microscopy images of A549 cells showing the colocalization of PDIA4 and caspase-3 in cells pretreated with Tm (50 ng/mL) for 3 days.

<u>Figure S6:</u> **(A)** Representative immunoblot showing PDIA4 levels in PDIA4-silenced A549 cells. **(B)** Representative immunoblot showing DNAJB12 and DNAJB14 levels in DNAJB12/14-silenced A549 cells. **(C-D)** Representative immunoblots showing SGTA levels in SGTA-silenced A549 cells. **(E)** Representative immunoblots showing cytosolic localization of PDIA4, PDIA1, and DNAJB11 in DNAJB12/14- or SGTA-silenced cells pretreated with 50ng/mL Tm or 7.5nM Tg. **(F-H)** Quantification of refluxed ER proteins DNAJB11, PDIA1, and PDIA4 corresponding to (E). **(I)** Representative immunoblot showing the interaction between Caspase-3, PDIA4, DNAJB12, DNAJB14, and SGTA in SGTA-silenced A549 cells pretreated with tunicamycin (Tm, 50 ng/mL) or thapsigargin (Tg, 7.5 nM) for 3 days. **(J)** Representative immunoblot showing the interaction between p53 and PDIA4 in A549 cells pretreated with Tm (50 ng/mL) or Tg (7.5 nM) for 3 days and subsequently challenged with high concentrations of cisplatin or doxorubicin. **(K)** Luciferase assay in PDIA4-silenced A549 cells pretreated with ER stressors, transfected with a p53-luciferase construct, and treated with 30μM cisplatin or 0.5μM doxorubicin. **(L)** Luciferase assay in SGTA-silenced or DNAJB12/14-silenced A549 cells pretreated with

ER stressors, transfected with a p53-luciferase construct, and treated with cisplatin or doxorubicin.

<u>Figure S7:</u> (A-E) Survival analysis of copy number alteration of PDIA4 in different types of cancer obtained from The Cancer Genome Atlas (TCGA) database [2]. (A) UCEC (Uterine Corpus Endometrial Carcinoma), (B) THCA (Thyroid carcinoma), (C) LGG (Low grade glioma), (D) MESO (Mesothelioma), and (E) PRAD (Prostate adenocarcinoma).

Supplementary References

[1] Gilbert LA, Horlbeck MA, Adamson B, Villalta JE, Chen Y, Whitehead EH, Guimaraes C, Panning B, Ploegh HL, Bassik MC, Qi LS, Kampmann M, Weissman JS. Genome-Scale CRISPR-Mediated Control of Gene Repression and Activation. Cell 2014;159. https://doi.org/10.1016/j.cell.2014.09.029.

[2] Smith JC, Sheltzer JM. Genome-wide identification and analysis of prognostic features in human cancers. Cell Rep 2022;38. https://doi.org/10.1016/j.celrep.2022.110569.
