# Supplementary figures and images for: Non-genetic inactivation of caspase-3 and P53 increases cancer cell fitness by PDIA4 redistribution
Source: Oncogene. 2025 Oct 21;44(47):4565–75. doi: 10.1038/s41388-025-03606-7 (PMC12623246; doi:10.1038/s41388-025-03606-7)

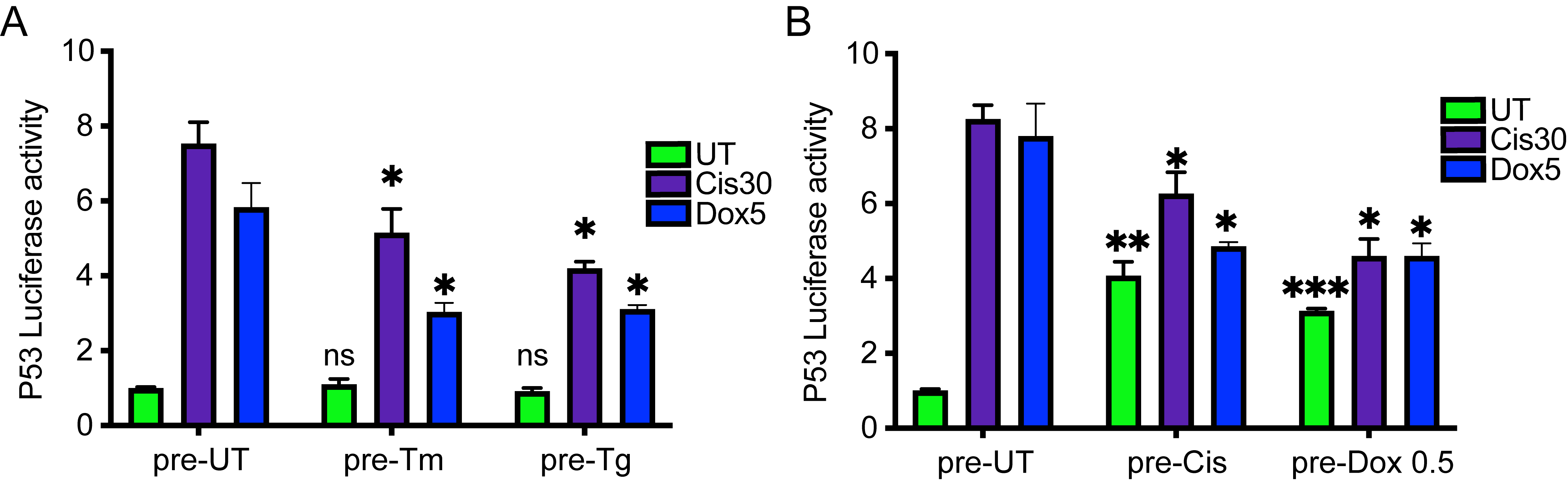

Supplement: Supplementary file 5 — SUPPLEMENTAL Figure S4 [file 41388_2025_3606_MOESM5_ESM.tif]
